# Generation of an inducible system to express polo-like kinase, Cdc5 as TAP fusion protein during meiosis in *Saccharomyces cerevisiae*

**DOI:** 10.1007/s13205-016-0503-x

**Published:** 2016-08-30

**Authors:** Rajni Vaid, Kamal Dev, Michael Lichten, Anuradha Sourirajan

**Affiliations:** 1 Faculty of Applied Sciences and Biotechnology, Shoolini University, Solan, Himachal Pradesh 173212 India; 2 Laboratory of Biochemistry and Molecular Biology, Center for Cancer Research, National Cancer Institute, Bethesda, MD 20892 USA

**Keywords:** Tandem affinity purification, Fusion protein, *Saccharomyces cerevisiae*, Meiosis, Polo-like kinase, Cdc5, Kinase dead, Estrogen inducible, Pachytene, Meiotic recombination, Joint molecules (JM), Substrates

## Abstract

Tandem affinity purification (TAP) is a highly efficient method for isolation of protein complexes from endogenous biological macromolecules. TAP system consists of dual affinity tags that facilitates the sequential purification of the desired proteins expressed at their low levels in vivo. Polo-like kinases (PLK) are serine/threonine protein kinases that are the crucial regulators of cell cycle. Cdc5, the solitary PLK in budding yeast *Saccharomyces cerevisiae*, has diverse array of targets in cell cycle. The present study was undertaken to construct an estrogen-inducible system for expression of Cdc5-TAP to isolate the substrates of Cdc5 during meiosis, particularly, pachytene stage of meiosis I. Two yeast strains were constructed *CDC5*-*IN* (*ndt80∆ pGAL1*-*CDC5*-*TAP*) and Cdc5-kinase inactive mutant (*ndt80∆ pGAL1*-*cdc5*-*N209A*-*TAP*). The estrogen-inducible expression of Cdc5-TAP and cdc5-N209A-TAP was validated by Western analysis. The systems would serve as a valuable tool for purification of substrates binding to Cdc5-TAP by TAP affinity chromatography.

**Electronic supplementary material:**

The online version of this article (doi:10.1007/s13205-016-0503-x) contains supplementary material, which is available to authorized users.

## Introduction

Polo-like kinases are serine threonine kinases characterized by the presence of protein kinase domain at the N-terminus, and a polo box domain (PBD) at the C-terminus, the latter playing a role in the modulation of kinase activity and substrate recognition (Lee et al. 2005). PLKs spatiotemporally regulate and coordinate mitosis and meiosis. A single PLK exists in *Saccharomyces cerevisiae* (Cdc5), while multiple PLKs have been identified in other organisms, except plants (Vaid et al. 2016). In *S. cerevisiae,* Cdc5, the solitary PLK regulates G2/M phase transition, metaphase to anaphase transition during mitosis, exit from mitosis and cytokinesis (Nigg 1998; Lee et al. 2005; Vaid et al. 2016). In eukaryotic systems, meiosis is imperative for genetic diversity and meiotic recombination. During meiosis, bi-phasic division takes place, wherein during the reductional division (meiosis I) homologous chromosomes segregate followed by equational division (meiosis II) producing four haploid gametes (Hochwagen and Amon 2006; Simchen 2009; Börner and Cha 2015). Cdc5 is a central regulator of meiosis I in *S. cerevisiae* (Attner et al. 2013; Vaid et al. 2016). Cdc5 regulates the meiotic functions in prophase I, including exit from pachytene, mono-orientation of the sister kinetochores, removal of cohesins from chromosomal arms at metaphase I and anaphase I, respectively, followed by meiotic divisions (Sharon and Simchen 1990; Clyne et al. 2003; Lee and Amon 2003a, b; Vaid et al. 2016). In *S. cerevisiae*, a meiosis-specific transcription factor, Ndt80, is essential for meiotic progression (Chu and Herskowitz 1998). Cells lacking Ndt80 arrest at pachytene and fail to complete meiotic divisions (Benjamin et al. 2003). During meiosis, transcription of *CDC5* is activated by Ndt80. Similar to Ndt80, Cdc5 has been shown to be necessary and sufficient for pachytene exit in meiosis I, raising the possibility of some targets of Cdc5 to be required for pachytene exit (Clyne et al. 2003; Lee and Amon 2003a, b; Sourirajan and Lichten 2008). However, the downstream targets of Cdc5 in pachytene exit have not been deciphered. Some of the targets of Cdc5 have been already identified, like Rec8, Mam1, and Ime2, none of which has a role in pachytene exit (Clyne et al. 2003; Lee et al. 2008; Sourirajan and Lichten 2008; Attner et al. 2013). There is still an array of unexplored targets, whose discovery could provide an insight into the role of Cdc5 in pachytene exit in specific, and meiosis as a whole.

The present study was initiated to develop an amenable tool for identification of the substrates of the PLK, Cdc5 during yeast meiosis. An estrogen-inducible system for Cdc5 expression during specific phase in meiosis was generated as described in previous studies (Benjamin et al. 2003; Sourirajan and Lichten 2008). The inducible system was combined with the TAP-tagging of Cdc5 to identify the targets of Cdc5 during yeast meiosis (Puig et al. 2001). The system was functionally validated by Western analysis.

Tandem affinity purification (TAP) tag is an affinity chromatography-based rapid and generic system of protein purification, which is further analyzed with mass spectrometry to identify the protein ensembles that are part of any functional proteome (Rigaut et al. 1999; Puig et al. 2001; Xu et al. 2010). The scope of TAP method in yeast allows the purification of TAP-tagged protein and its associated components using dual affinity system (Rigaut et al. 1999). The C-terminal TAP insertion cassette consists of two domains, proximally a calmodulin-binding peptide, a tobacco etch virus (TEV) protease cleavage site (Dougherty et al. 1989; Kapust et al. 2001) in the center and an IgG binding domain of *Staphylococcus aureus* protein A at the distal end (Uhlen et al. 1983; Puig et al. 2001). The desired protein with C-terminal TAP tag is sequentially purified under mild conditions on IgG matrix followed by the elution with TEV protease, and then passed through the calmodulin resin in the presence of Ca^2+^ ions and finally eluting the protein of interest in presence of ethylene glycol tetra acetic acid (EGTA) (Puig et al. 2001; Van Driessche et al. 2005).

## Methods

Chemicals and reagents of biochemical and molecular biology grade were procured from Himedia Labs, India; MP Biomedicals, USA; Fermentas Inc. USA; Bio-Rad, USA. Antibodies used in this study were purchased from Thermo scientific, USA and Santa Cruz Biotechnologies, Inc. USA.

### Strains and plasmids

All the *S. cerevisiae* strains used in this study were of SK1 genotype (Kane and Roth 1974; Allers and Lichten 2001). The genomic-tagged yeast strains containing *CDC5*-*TAP* (with C-terminus TAP tag) in the SK1 background were a kind gift of Dr. Wolfgang Zachariae, Max Planck Institute of Molecular Cell Biology and Genetics, Germany (Matos et al. 2008). The *CDC5*-*TAP* yeast strains containing the *URA3*-*ARG4* recombinant inserts were constructed using genetic crosses. The genotypes of the yeast strains used in this study are given the Table S1 (Supplementary material).

Two integrant plasmids pMJ830 and pMJ840 were used to generate *CDC5*-*IN* and *cdc5*-*N209A*-*IN* yeast strains (Supplementary material). *S. cerevisiae* strains were grown on YEPD medium (1 % yeast extract, 2 % peptone, and 2 % dextrose), supplemented with 0.8 % adenine sulfate at 30 °C. Nutrient broth (NB, Himedia Labs, Mumbai) with 100 μg/ml ampicillin was used to culture and grow the bacterial strains of *E. coli* DH5α harboring pMJ830 and pMJ840 at 37 °C. Plasmid DNA of pMJ830 and pMJ840 were isolated and digested with SnaB I at 37 °C for 1 h and used in transformations. Transformants were grown and selected on hygromycin selection medium (YEPD medium containing 300 μg/ml of hygromycin) at 30 °C.

## Construction of *CDC5*-*IN* (*ndt80∆ pGAL1*-*CDC5*-*TAP*) and *cdc5*-*N209A*-*IN* (*ndt80∆ pGAL1*-*cdc5N209A*-*TAP*) diploid strains

### Transformation of the yeast strain with SnaB I digested pMJ830 and pMJ840

Construction of *CDC5*-*IN* and *cdc5*-*N209A*-*IN* diploid strains was done through genomic integration approach. 5 ml of YEPD broth was inoculated with single colony of the haploid parental yeast strain S3561 (Table S1) and cultured at 30 °C for 16 h. 0.5 ml of the culture (*A*_600_ ~2.0) of S3561 was harvested and the yeast cells were transformed with two microgram of each of the SnaB I digested and linearized plasmids of pMJ830 and pMJ840 using EZ-transformation method (MP Biomedicals, USA). The transformation mixtures were plated on hygromycin selection media (YEPD containing 300 µg/ml hygromycin) and incubated at 30 °C for 2–3 days until transformant colonies were observed.

### Verification of the transformants

The integration of the plasmid at the *CDC5* locus was verified by PCR reactions using gene specific and vector specific primers (Table S2). The DNA of the haploid transformants was isolated (Sambrook et al. 2009) and the PCR was set with three different primer sets as111f–as113r, as65f–as113r, and as65f–as106r, respectively. The sequence of each primer is given in Table S2 (Supplementary material). The reaction mixture of PCR contained Dream Taq (2U), reaction buffer 1X, dNTP mix (100 µM each), primers (1 µM each). PCR was run at 94 °C, 2 min (initial denaturation); 35 cycles each of denaturation, annealing, and extension; at 94 °C, 2 min; 58 °C, 30 s, and 72 °C, 2 min 30 s, respectively, with a final extension of 72 °C, 10 min.

### Generation of ASd1 (CDC5-IN) and ASd2 (cdc5-N209-IN) diploids

The resulting *CDC5*-*TAP*-*IN* (ASh1) and *cdc5*-*N209A*-*TAP* (ASh2) haploid transformant strains after confirming by PCR were crossed with the other wild-type parental strain of opposite mating type (Mat α; S3565) on YEPD plates to result in *CDC5*-*TAP*-*IN* (ASd1) and *cdc5*-*N209A*-*TAP*-*IN* (ASd2) diploid strains, respectively. The genotype of the strains is described in Table S1 (Supplementary material).

### Inducible expression of *Cdc5*-*TAP* in meiosis

#### Induction of meiosis (Sporulation)

The *CDC5*-*IN* and *cdc5*-*N209A*-*IN* diploid strains were first grown in YEPD medium supplemented with 0.8 % adenine sulfate at 30 °C for 24 h. For induction of meiosis, yeast cells were subjected to nitrogen deprivation by transferring to 1 % potassium acetate at 30 °C with vigorous shaking at 350 rpm, as described (Goyon and Lichten 1993; Allers and Lichten 2001). The culture was incubated for 7 h at 30 °C (by when most of the cells will complete pachytene and arrest at pachytene stage, owing to the absence of Ndt80) (Allers and Lichten 2001; Sourirajan and Lichten 2008). After 7 h, Cdc5-TAP expression was induced in a portion of the meiotic culture by the addition of 1 µM β-estradiol (ED). 5–10 ml of meiotic yeast cells were collected from uninduced samples and after 2 h past induction for western analysis.

### Preparation of protein extracts and Western blotting

Proteins were extracted using 20 % tri-chloroacetic acid (TCA) and estimated by Bradford method (Bradford 1976). The protein extracts (15 µg of total protein) were resolved on 10 % SDS–polyacrylamide gel and electroblotted onto the nitrocellulose membrane. The blot was incubated in blocking solution containing 5 % non-fat dried milk for 1 h. Membrane was then incubated with 1:1000 dilution of rabbit anti-TAP tag antibodies (Thermo Scientific, USA) overnight at 4 °C. Membrane was then incubated with 1:7500 dilution of anti-rabbit IgG conjugated to horseradish peroxidase (Santa Cruz Biotechnologies, USA) for 1 h and developed using enhanced chemiluminescence kit (ECL, Bio-Rad, Inc. USA).

## Results and discussion

### Generation of an estradiol-inducible *CDC5*-*TAP* expression system to purify the substrates of Cdc5 during meiosis

**Fig. 1:**
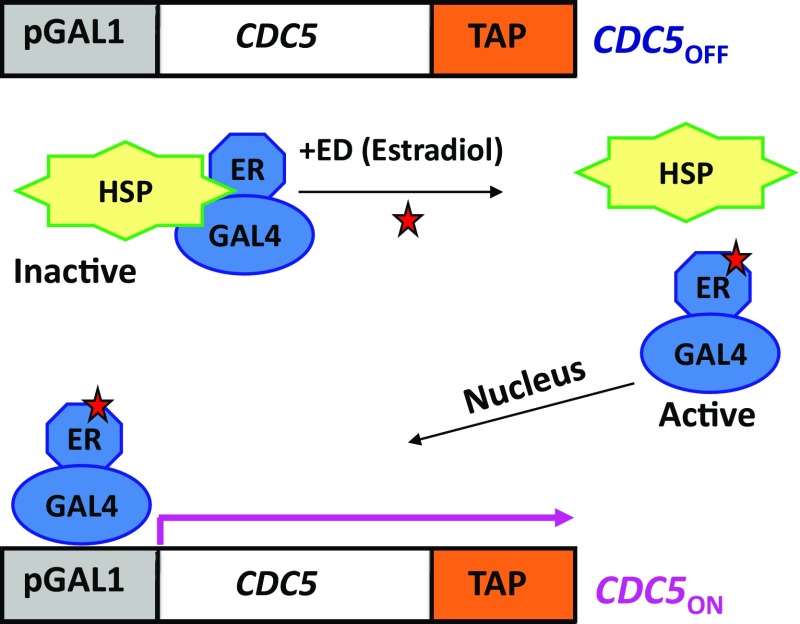
Estrogen-inducible system to achieve Ndt80-independent expression of *CDC5*-*TAP* during meiosis. In the absence of the ligand, estradiol (ED), *CDC5*-*TAP* gene is off. Addition of ED activates Gal4:ER, which translocates to nucleus and turns on *CDC5* expression from *pGAL1* promoter

The experimental system consists of the inducible and Ndt80-independent expression of *CDC5*-*TAP* in *ndt80*Δ cells during meiosis (Sourirajan and Lichten 2008). Yeast mutants lacking NDT80 (*ndt80*Δ) arrest at pachytene (Xu et al. 1995), thereby serving as a synchronous population of meiotic cells in pachytene stage. The *CDC5* gene consists of tandem affinity purification (TAP) tag at the end of the gene, which will produce a Cdc5-TAP protein with a TAP tag at the C-terminus. The TAP tag will facilitate the purification of Cdc5-bound substrates in meiosis. Expression of *CDC5*-*TAP* from *pGAL1* is achieved using a chimeric Gal4:estrogen receptor (ER) transcription factor (Gal4:ER), which in turn, is activated by the addition of the ligand inducer, estradiol (ED) (Fig. 1).

### Construction of yeast strains for inducible expression of Cdc5-TAP and cdc5-N209A-TAP during meiosis

**Fig. 2:**
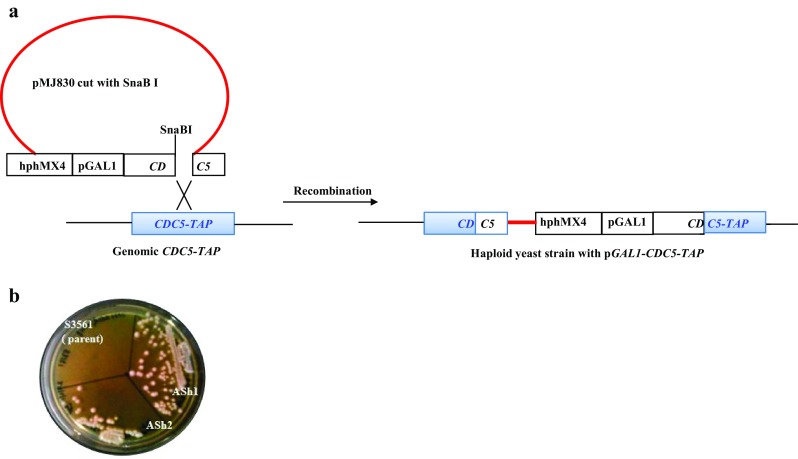
**a** Schematic representation of the strategy used for construction of *CDC5*-*TAP*-*IN* strain by integration of SnaB I digested pMJ830 plasmid containing *pGALl*-*CDC5* in the yeast cells (details in the text). **b** Growth of transformants ASh1 (*pGAL1*-*CDC5*-*TAP)* and ASh2 (*pGAL1*-*cdc5*-*N209A*-*TAP)* on hygromycin selection medium. The untransformed S3561 parent showed no growth

To construct the *CDC5*-*IN* (*ndt80∆ pGAL1*-*CDC5*-*TAP*) diploid strain, genomic integration approach was adopted. In the current strategy, one of the haploid parental strains (S3561) containing *CDC5*-*TAP* was transformed with SnaB I linearised integrant plasmid, containing *pGAL1*-*CDC5* (pMJ830). The plasmid also contains *hphMX4* marker cassette, which encodes for resistance to hygromycin and thus, the transformants could be selected by plating on hygromycin media (Fig. 2a). The homologous recombination of the integrant plasmid with the genomic *CDC5*-*TAP* resulted in the generation of haploid parental strain ASh1, with tandem copies of *CDC5* gene, one under its own promoter and the *CDC5*-*TAP* under *pGAL1* promoter (Fig. 2a).

An additional strain *cdc5*-*N209A*-*TAP*-*IN* (kinase-dead inducible strain) was constructed using the same strategy as described above, except for the transformation with linearised integrant plasmid with SnaB I enzyme, containing *pGAL1*-*cdc5*-*N209A* (pMJ840) (Fig. 2). The rationale for the construction of the kinase-dead version of the *CDC5*-*IN* strain was based on the fact that the interaction of catalytically inactive Cdc5 kinase (cdc5-N209A) with the substrate/s will be long-lived compared to its wild-type counterpart kinase enzyme, thereby enhancing the efficiency of co-purification of substrates of Cdc5 kinase during TAP-purification. After 3 days of incubation, isolated colonies of transformants were observed (~20 per plate). On the other hand, no growth was observed in plates with untransformed parental host strain (S3561) (Fig. 2b). About 8–10 colonies of the transformants were picked and further purified by two rounds of subculturing on the hygromycin selection media. The transformants were selected and used for subsequent experiments.

### PCR confirmation of transformants

**Fig. 3:**
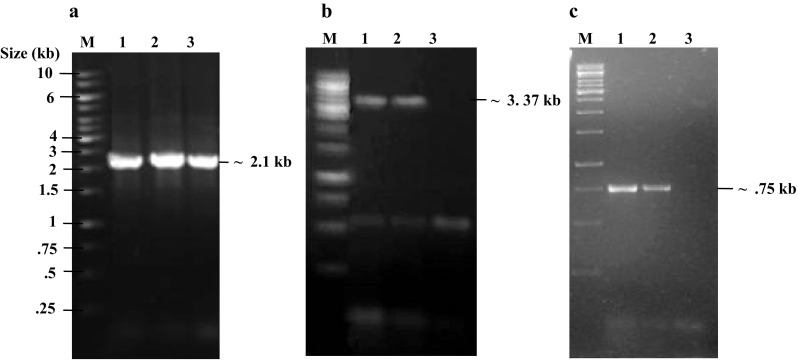
Confirmation of *Cdc5*-*TAP*-*IN* and *cdc5*-*N209A*-*IN* transformants by PCR. Forward primers as111f, as65f, and reverse primers as106r, 113r, respectively, were used for the verification of *CDC5*-*IN* and *cdc5*-*N209A*-IN transformants by PCR. **a** PCR verification with primer set as111f – as113r. *M* size marker (1 kb ladder); *lanes 1*, *2*, *3* S3561 (wild-type), ASh1 and ASh2 (transformants), respectively, show (~2.1 kb) PCR products as expected for wild type and transformants. **b**, **c** PCR Confirmation of transformants with primer sets as65f–as106r and as65f–as113r, respectively. *M* size marker (1 kb ladder), *Lanes 1*, *2* ASh1 and ASh2, respectively; *lane 3* S3561 (wild-type). In **b**, **c** both transformants ASh1 and ASh2 show PCR products of expected size of ~3.37 and ~0.75 kb, respectively, whereas, no PCR product was obtained for the untransformed wild-type S3561

The transformants Ash1 and Ash2 were confirmed by PCR with three different sets of primers to verify the plasmid integration of *pGAL1*-*CDC5*-*TAP* (pMJ830) and *pGAL1*-*cdc5*-*N209*-*TAP* (pMJ840) as described in the “Methods”. As expected, the primer set as111f–as113r produced a PCR product of ~2.1 kb for the wild type, ASh1 and ASh2 (transformants) (Fig. 3a). The primer sets as65f–as113r and as65f–as106r amplified products of ~3.3 kb and 750 bp, respectively, in the transformants, whereas no amplification was observed in the untransformed yeast strain, S3561, with either of the primer sets (Fig. 3b, c). Both ASh1 and ASh2 strains were crossed with the other parental strain of opposite mating type (S3565), which resulted in the diploid strains ASd1 (*CDC5*-*TAP*-*IN*) and ASd2 (*cdc5*-*N209A*-*TAP*-*IN*) (Table S1, Supplementary material).

### Inducible expression of Cdc5-TAP and cdc5-N209A-TAP

**Fig. 4:**
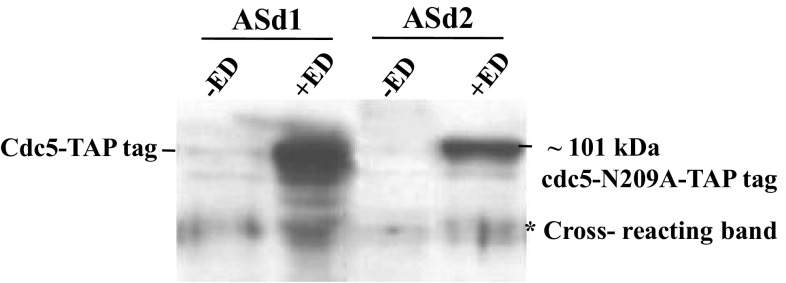
Verification of inducible expression of Cdc5-TAP and cdc5-N209A-TAP kinase-dead proteins in meiotic cultures. Western analysis of the meiotic protein extracts of ASd1 and ASd2 in the absence (−ED) and presence (+ED) of inducer with anti-TAP antibodies. Samples were collected after 2 h of induction. A specific band corresponding to the size of Cdc5-TAP or cdc5-N209A-TAP fusion proteins (~101 kDa) is observed upon ED addition

The diploids *CDC5*-*TAP*-*IN* and *cdc5*-*N209A*-*TAP*-*IN* were incubated in sporulation medium to induce meiosis for 7 h, followed by the addition of inducer (ED). Inducible expression of CDC5-TAP and cdc5-N209A-TAP was confirmed by Western analysis using anti-TAP tag antibodies (Fig. 4). On the other hand, the expression of Cdc5-TAP or cdc5-N209A-TAP was absent in the uninduced samples (−ED) (Fig. 4).

## Conclusion

In our study, we have successfully generated *S. cerevisiae* strains that were engineered within an inducible Cdc5-TAP construct for the purification of substrates binding Cdc5 kinase. The inducible expression of *CDC5*-*TAP* and its kinase-dead version system was validated by western analysis. Thus, in our study we have constructed *ndt80∆ CDC5*-*IN (pGAL1*-*CDC5*-*TAP)* strain to induce and express target protein during pachytene of meiosis-I.

The TAP-tagging *CDC5* would provide a scope to aim the targets that are phosphorylated during pachytene exit. Thus, in future, the Cdc5-IN system will be used to pull-down the substrates of Cdc5 during meiosis. The approach can be extended to study other cellular processes in yeast meiosis. This approach shall provide opportunity to study organizational, functional, and interaction patterns of proteome involved during pachytene exit. In future, the combination of estradiol-inducible expression and TAP-tag systems could be used to analyze the proteome during specific stages of meiosis.

## Electronic supplementary material

Below is the link to the electronic supplementary material.

Supplementary material 1 (DOCX 17 kb)
