# Supplementary material for: Generation of an inducible system to express polo-like kinase, Cdc5 as TAP fusion protein during meiosis in Saccharomyces cerevisiae
Source: 3 Biotech. 2016 Aug 30;6(2):185. doi: 10.1007/s13205-016-0503-x (PMC5005230; doi:10.1007/s13205-016-0503-x)
Supplement: Supplementary file 1 — Supplementary material 1 (DOCX 17 kb) [file 13205_2016_503_MOESM1_ESM.docx]

**Supplementary material**

***Construction of yeast strains***

All the yeast strains used in the study were of SK1 background (Kane and Roth, 1974). Haploid parents of the diploid yeast strains used here were derived by transformation of the haploid strain S3561 (Table S1) with SnaB I cut plasmid pMJ830 or pMJ840. The transformants of S3561 (ASh1 and ASh2) were crossed with wild-type strain (S3565) of opposite mating type for construction of diploids ASd1 (*CDC5-TAP-IN)* and Asd2 (*CDC5-N209A-TAP-IN)*. The genotype of all these strains is described in Table S1. The details of *pGPD1-GAL4 (848) ER::URA3* construct are described in Sourirajan and Lichten (2008). S3561 and S3565 strains were constructed by AS by genetic crosses in the laboratory of Dr. Michael Lichten, NCI, NIH, USA. Both the parents harbor an *URA3* and *ARG4* inserts at the *HIS4* (S3561) and *LEU2* (S3565) loci, respectively (Sourirajan and Lichten, 2008). The *NDT80* gene is disrupted and deleted by insertion of KanMX, encoding kanamycin resistance.

**Table S1. Genotype of yeast strains used in the study**

| **Strain** | **Parent** | **Relevant Genotype** |
| --- | --- | --- |
| S3561 |  | *MATa ura3 lys2 ho::LYS2 arg4∆ his4::URA3rev-tel-arg4-ecPal9 pac1(62528) Sph1 trp1::hisG CDC5-TAP-KlTRP1 ndt80∆::KanMX* |
| S3565 |  | *MATα trp1::hisG ura3::pGPD1-GAL4(848).ER::URA3 lys2 ho::LYS2 arg4∆*  *leu2-R::URA3 tel-ARG4 ndt80∆::KanMX CDC5-TAP-KlTRP1* |
| ASh1 | S3561/ pMJ830 | *MATa ura3 lys2 ho::LYS2 arg4∆ his4::URA3rev-tel-arg4-ecPal9pac1(62528)-Sph1 trp1::hisG ndt80∆::KanMX pCDC5-CDC5-pFa6a-HphMX4- pGAL1-CDC5-TAP-KlTRP1* |
| ASh2 | S3561/ pMJ840 | *MATa ura3 lys2 ho::LYS2 arg4∆ his4::URA3rev-tel-arg4-ecPal9pac1(62528)-Sph1 trp1::hisG ndt80∆::KanMX pCDC5- CDC5 -pFa6a-HphMX4- pGAL1- cdc5-N209A -TAP-KlTRP1* |

***Plasmids used in the study***

pMJ830 (*pGAL1-CDC5*) was constructed as described (De Muyt et al. 2012) and pMJ840 (*pGAL1-cdc5-N209A*) was constructed in the similar manner and both plasmids used in the study were ~ 7.5 kb in size. The plasmid inserts contain *hphMX4* marker cassette, which encodes for resistance to hygromycin and thus, the transformants, can be selected by plating on hygromycin containing media.

***Primers used in the study***

The sequence of the primers used in the study are given in Table S2.

**Table S2.**

| **Primer** | **Sequence (5’ to 3’)** | **Nucleotides** |
| --- | --- | --- |
| **as111f** | GTTATCAACAGAGGATATTGGTGTC | 25 |
| **as113r** | CCAATCAATGGACTGGTAATTTCG | 24 |
| **as65f** | GGGTAATTAATCAGCGAAGCGATG | 24 |
| **as106r** | TTCTGGTTCTGTTAAAACTTTTCTTC | 26 |

**References:**

Kane SM, Roth R (1974) Carbohydrate metabolism during ascospore development in yeast. J Bacteriol 118: 8-14

Sourirajan A, Lichten M (2008) Polo-like kinase Cdc5 drives exit from pachytene during budding yeast meiosis. Genes Dev 22: 2627-2632

De Muyt A, Jessop L, Kolar E et al (2012) BLM helicase ortholog Sgs1 is a central regulator of meiotic recombination intermediate metabolism. Mol Cell 46: 43-53
